# Implementing Mitigation Strategies in Early Care and Education Settings for Prevention of SARS-CoV-2 Transmission — Eight States, September–October 2020

**DOI:** 10.15585/mmwr.mm6949e3

**Published:** 2020-12-11

**Authors:** Fátima Coronado, Sara Blough, Deborah Bergeron, Krista Proia, Erin Sauber-Schatz, Marco Beltran, Katherine Troy Rau, Andria McMichael, Tracye Fortin, Mark Lackey, Jovanna Rohs, Tracey Sparrow, Grant Baldwin

**Affiliations:** ^1^CDC COVID-19 Response Team; ^2^Office of Head Start, Washington, DC; ^3^Educare Learning Network, Chicago, Illinois.

The Head Start program, including Head Start for children aged 3–5 years and Early Head Start for infants, toddlers, and pregnant women, promotes early learning and healthy development among children aged 0–5 years whose families meet the annually adjusted Federal Poverty Guidelines* throughout the United States.^†^ These programs are funded by grants administered by the U.S. Department of Health and Human Services’ Administration for Children and Families (ACF). In March 2020, Congress passed the Coronavirus Aid, Relief, and Economic Security (CARES) Act,^§^ which appropriated $750 million for Head Start, equating to approximately $875 in CARES Act funds per enrolled child. In response to the coronavirus disease 2019 (COVID-19) pandemic, most states required all schools (K-12) to close or transition to virtual learning. The Office of Head Start gave its local programs that remained open the flexibility to use CARES Act funds to implement CDC-recommended guidance (*1*) and other ancillary measures to provide in-person services in the early phases of community transmission of SARS-CoV-2, the virus that causes COVID-19, in April and May 2020, when many similar programs remained closed. Guidance included information on masks, other personal protective equipment, physical setup, supplies necessary for maintaining healthy environments and operations, and the need for additional staff members to ensure small class sizes. Head Start programs successfully implemented CDC-recommended mitigation strategies and supported other practices that helped to prevent SARS-CoV-2 transmission among children and staff members. CDC conducted a mixed-methods analysis to document these approaches and inform implementation of mitigation strategies in other child care settings. Implementing and monitoring adherence to recommended mitigation strategies reduces risk for COVID-19 transmission in child care settings. These approaches could be applied to other early care and education settings that remain open for in-person learning and potentially reduce SARS-CoV-2 transmission.

In collaboration with ACF, CDC conducted a mixed-methods study during September–October 2020 in Head Start programs in eight states (Alaska, Georgia, Idaho, Maine, Missouri, Texas, Washington, and Wisconsin). Head Start programs, each with five to 17 centers and 500–2,500 children, were selected by the Office of Head Start. The four-phase study design included reviews of standard operating procedures (SOPs) for COVID-19 mitigation, deployment of an online survey for program directors to document mitigation strategies implemented and COVID-19 cases reported, in-depth interviews with staff members from five programs overall, and observation of mitigation strategy implementation during a virtual visit to one Head Start site. This activity was reviewed by CDC and was conducted consistent with applicable federal law and CDC policy.^¶^

All program sites closed for periods ranging from 2 weeks to 2 months after state-initiated mandates in April and May and upon reopening offered a hybrid** learning model (i.e., in-person and virtual). The Office of Head Start allowed administrative flexibility in how programs could use funding, encouraged innovation in implementing CDC guidance (*1*), and provided resources for implementing multiple concurrent preventive strategies (e.g., delivery of webinars to >240,000 staff members, parents, community members, and partners). All programs developed SOPs during March–April 2020 and began implementing these procedures in April. All SOPs covered multicomponent mitigation practices and promoted behaviors designed to reduce infection spread, create healthy environments, facilitate healthy operations, and explain procedures to follow in the event of identification of a COVID-19 case.

Seven of eight Head Start programs, representing 55 centers, responded to the survey. All reported implementing SOPs and adjusting them depending on guidance from the local public health authorities or education department, local level of transmission and related factors described below. Multiple strategies were implemented simultaneously, including training teachers and encouraging caretakers to adhere to SOPs and mitigation strategies; instituting flexible medical leave policies for staff members; providing and requiring use of masks for all staff members and children; and supervising handwashing and hand-sanitizing for children (Box). Variations regarding methods for screening the health of staff members and children were noted; among these methods, self-administered temperature checks upon arrival were most frequently reported for staff members. Screening for signs and symptoms^††^ of illness upon arrival was most frequently reported for children. Mask policies for children varied, and exemptions for children aged <2 years and those with special health care and education needs were allowed. All programs reported increased cleaning and disinfecting or sanitizing of high-traffic areas, high-touch surfaces, and toys. Five programs reported increasing cleaning and disinfecting of bedding and improving ventilation. Guidance from public health or education agencies and state or local mandates were the factors most commonly reported to influence decisions about SOP adjustments. Other, less frequently reported, factors included concerns about transmission of SARS-CoV-2 within facilities and perceived pressure from the community.

BOX COVID-19 mitigation strategies implemented by Head Start and Early Head Start child care programs — eight states,* September–October 2020

**Everyday prevention actions**

- Reinforcement of hand hygiene behavior and respiratory etiquette
- Supervised handwashing and hand-sanitizing for children
- Intensified cleaning and disinfection efforts (e.g., with toys, frequently touched surfaces, and bedding)
- Required use of masks for staff members, visitors, and children aged >2 years
- Social distancing to the extent possible
- Daily health screening procedures on arrival for children and staff members
- Drop-off and pick-up procedures
- Monitoring for absenteeism
- Ability to monitor and restock supplies
- Steps to increase ventilation including installation of ion air purifiers
- Steps to decrease occupancy in areas without increased ventilation
- Use of outdoor space as much as possible
- Cohorting by classroom to minimize exposure between groups

**Actions when someone is ill**

- COVID-19 point of contact identified
- Staff members trained in COVID-19 safety protocols
- Requiring ill children and staff members to stay at home
- Vigilance for symptoms
- Daily screening of staff members and children for signs and symptoms before facility entry
- Standard operating procedures for when a child or staff member experiences symptoms
- Identification of isolation room
- Plan to notify local health official of COVID-19 cases
- Plan to distribute instructions for primary care referral, testing, or both
- Plan to distribute instructions or guidance for home isolation
- Plan to require close contacts to wait 14 days before returning
- Flexible COVID-19 medical leave policies for staff members

**Communications and support**

- Training and ongoing reinforcing of standard operating procedures and mitigation measures with caregivers, teachers, and other staff members
- Vigilance and training for the identification of COVID-19 related symptoms
- Masks and other personal protective equipment (e.g., face shields and gowns) provided to teachers and other staff members
- Incentives to adhere to mitigation strategies
- Flexible medical leave policies for staff members with emphasis on persons at higher risk for severe illness and those with caregiving responsibilities
- Flexible work hours and staggered shifts
- Telework options for staff members at higher risk for severe illness

**Abbreviation:** COVID-19 = coronavirus disease 2019.

* Alaska, Georgia, Idaho, Maine, Missouri, Texas, Washington, and Wisconsin.

All programs reported having plans in place for managing children and staff members experiencing COVID-19 symptoms. Three programs identified nine cases among children in three centers (range = one to four cases per center) during May and June. Administrators followed SOPs for notification, isolation, facility closure, and cleaning and disinfection. All three centers were closed for in-person operation for 14 days after identification of a case but offered virtual options to continue providing services. Respondents from all seven programs reported that centers had a designated isolation area. One program did not report whether a designated isolation area existed; however, this program reported ability to isolate a suspected case. All but one program had a protocol for working with the local health department if a positive case was identified; all indicated that the local health department would be contacted if a case was identified.​ All programs had established procedures for notifying parents or caregivers of close contacts.

Interviews were conducted in September and October with program directors identified by the Office of Head Start in five states (Alaska, Georgia, Maine, Missouri, and Wisconsin). A common theme identified was the flexibility offered for staffing and operations, including flexible medical leave, enhanced benefits during the pandemic (e.g., additional financial benefits to cover health care–associated costs), and remote working options. Staff members who were at increased risk for severe illness^§§^ because of underlying medical conditions or age and those with caregiving responsibilities were offered virtual and hybrid teaching opportunities, flexible hours, and staggered shifts. Policies were put into place for staff members to stay at home without fear of job loss or other consequences. In addition to providing personal protective equipment (e.g., gloves and masks), staff members were furnished with cleaning and other supplies and were offered training, ongoing reinforcement of SOPs, and incentives to abide by mitigation strategies (e.g., a program provided a financial incentive for staff members to purchase additional supplies).

A second theme identified was ongoing communications among program administrators, parents and caregivers, and teachers and other staff members to ensure understanding of SOPs. Communications included updates on program websites, development of instructional videos, written information, virtual meetings, media coverage, social media postings, and posted signage at facilities.

Factors facilitating successful implementation of mitigation strategies included extensive communication with consistent messaging to staff members and parents; ongoing training and support to staff members; continuous engagement of community partners and parents; and collaboration with program nurses, local health departments, hospital systems, and community organizations (e.g., United Way and Boys & Girls Club). Challenges included maintaining recommended social distancing, ventilation, weather concerns during the fall and heading into winter, parental mental health concerns (e.g., chronic stress, depression, anxiety, and trauma related to losing a loved one to COVID-19), questions concerning effects of staff members wearing masks on infant and toddler psychosocial development, maintaining guidance vigilance, and concern that programs were being overly cautious.

A virtual visit to a Head Start site in Texas found that staff members and children observed social distancing. In rooms with children aged <2 years, mask use was only observed among staff members, per CDC guidelines. Physical dividers were observed, including an innovative playground divider purchased with CARES Act funding that allowed for more outdoor play time for children. Cleaning and disinfecting protocols were described, along with guidance for stocking and monitoring the supply room to ensure adequate supplies. Plans for responding to positive SARS-CoV-2 tests were reviewed, and all included a rapid notification system for all enrolled families. To continue enrollment for the fall, a contactless application system using quick-response codes on community flyers had been implemented.

## Discussion

Children can acquire and transmit SARS-CoV-2 in school and child care settings (*2*,*3*). Since the COVID-19 pandemic started, Head Start and Early Head Start programs successfully implemented CDC-recommended mitigation strategies and applied other innovative approaches to limit SARS-CoV-2 transmission among children, teachers, and other staff members by allowing maximum program flexibility and allocating financial and human resources. As CDC learned more about COVID-19, the agency provided updated guidance for various settings, including child care programs, with options for screening children upon arrival. This guidance helps to ensure that children who have a fever or other signs of illness are not admitted to the facility and offers additional options that can be considered if personal protective equipment is in short supply (*1*).

SARS-CoV-2 transmission investigations in Rhode Island and Utah indicated that implementation of CDC-recommended mitigation strategies contributed to limiting transmission of SARS-CoV-2 in child care facilities in both states (*3*,*4*). This report describes how a comprehensive, multipronged approach for SARS-CoV-2 mitigation strategies, used in early care and education settings, specifically Head Start programs, might have helped to slow transmission, as few cases occurred. Financial and staffing resources were allocated to prioritize mitigation strategies; support to staff members and parents were critical components for these programs to help minimize the potential for negative consequences that can be associated with child care center closure, including providers’ loss of jobs and wages, parents’ challenges when returning to work, and children’s diminished educational, social, and nutritional opportunities (*5*).

Implementing and monitoring adherence to CDC-recommended mitigation strategies could play a crucial role in reducing SARS-CoV-2 transmission in child care settings. CDC developed tools and resources for child care programs, including examples of evaluation questions, related qualitative and quantitative indicators, and suggested data sources to understand the impact of COVID-19 mitigation strategies in child care programs (*6*). For example, child care facilities can identify facilitators, barriers, and other factors affecting implementation of mitigation strategies. Baseline information can include characteristics of the child care program (e.g., number of children in the program, child-to-staff member ratio, parental or community attitudes and involvement, and rates of retention or attrition among staff members and volunteers). This can help identify gaps and areas where additional mitigation strategies can be implemented or strengthened.

The findings in this report are subject to at least two limitations. First, this qualitative descriptive analysis might not be generalizable beyond the participating Head Start programs; however, programs were geographically diverse and represented all four U.S. Census regions. Second, study outcomes could not be attributed to implemented mitigation strategies; however, these strategies and the merits of a multicomponent mitigation approach have been documented to reduce SARS-CoV-2 transmission (*7*,*8*). Additional evaluation is needed to understand how multicomponent mitigation strategies work in child care settings that remain open for in-person learning in areas with high community transmission.

The benefits of child care programs (e.g., helping to achieve developmental milestones, nutritional support, socialization, and improved mental health) are many. Understanding child care programs’ capabilities for implementing COVID-19 mitigation strategies provides practical information that public health officials, child care setting administrators, and evaluators can use to implement and adjust strategies to reduce SARS-CoV-2 transmission. Child care settings should implement concurrent preventive measures and adjust these strategies based on community transmission data (*9*).

**Summary**

**What is already known about this topic?**

The benefits of in-person child care programs are myriad; however, SARS-CoV-2 transmission has been documented in child care facilities.

**What is added by this report?**

Head Start and Early Head Start programs successfully implemented CDC-recommended guidance and other ancillary measures for child care programs that remained open, allowing them to continue offering in-person learning. These approaches were documented to guide implementation of mitigation strategies in child care settings.

**What are the implications for public health practice?**

Implementing and monitoring adherence to recommended mitigation strategies can reduce risk for SARS-CoV-2 transmission in child care settings. These approaches could be applied to other early care and education settings that remain open for in-person learning and potentially reduce the spread of coronavirus disease 2019.

## Footnotes

* https://aspe.hhs.gov/poverty-guidelines.

† https://www.acf.hhs.gov/ohs/about/head-start.

§ https://home.treasury.gov/policy-issues/cares.

¶ 45 C.F.R. part 46, 21 C.F.R. part 56; 42 U.S.C. Sect. 241(d); 5 U.S.C. Sect. 552a; 44 U.S.C. Sect. 501 et seq.

** https://www.cdc.gov/coronavirus/2019-ncov/community/schools-childcare/schools.html.

†† https://www.cdc.gov/coronavirus/2019-ncov/symptoms-testing/symptoms.html.

§§ https://www.cdc.gov/coronavirus/2019-ncov/need-extra-precautions/people-with-medical-conditions.html.
